# Oxidative stress is involved in LLLT mechanism of action on skin healing in rats

**DOI:** 10.1590/1414-431X202010293

**Published:** 2021-04-26

**Authors:** D.D. Hartmann, R.P. Martins, T.C. da Silva, S.T. Stefanello, A.A. Courtes, D.F. Gonçalves, A.B.V. Furtado, B.S.L. Duarte, L.U. Signori, F.A.A. Soares, G.O. Puntel

**Affiliations:** 1 Centro de Ciências Naturais e Exatas, Programa de Pós-Graduação em Ciências Biológicas e Bioquímica Toxicológica, Universidade Federal de Santa Maria, Santa Maria, RS, Brasil; 2 Centro de Ciências da Saúde, Departamento de Fisioterapia, Universidade Federal de Santa Maria, Santa Maria, RS, Brasil; 3 Centro de Ciências Naturais e Exatas, Departamento de Bioquímica e Biologia Molecular, Universidade Federal de Santa Maria, Santa Maria, RS, Brasil; 4 Centro de Ciências da Saúde, Departamento de Morfologia, Universidade Federal de Santa Maria, Santa Maria, RS, Brasil

**Keywords:** Rehabilitation, Phototherapy, LLLT, Skin tissue, Inflammation

## Abstract

The skin injury healing process involves the main phases of homoeostasis, inflammation, proliferation, and remodeling. The present study aimed to analyze the effects of low-level laser therapy (LLLT) on hematological dynamics, oxidative stress markers, and its relation with tissue healing following skin injury. Wistar rats were divided into control, sham, skin injury, and skin injury LLLT. The biochemical and morphological analyses were performed in the inflammatory (1 and 3 days) and regenerative phases (7, 14, and 21 days) following injury. The skin injury was performed in the dorsal region, between the intrascapular lines, using a surgical punch. LLLT (Al-Ga-In-P, λ=660 nm, energy density of 20 J/cm^2^, 30 mW power, and a time of 40 s) was applied at the area immediately after injury and on every following day according to the experimental subgroups. LLLT maintained hematocrit and hemoglobin levels until the 3rd day of treatment. Surprisingly, LLLT increased total leukocytes levels compared to control until the 3rd day. The effects of LLLT on mitochondrial activity were demonstrated by the significant increase in MTT levels in both inflammatory and regenerative phases (from the 1st to the 7th day), but only when associated with skin injury. The results indicated that LLLT modulated the inflammatory response intensity and accelerated skin tissue healing by a mechanism that involved oxidative damage reduction mostly at early stages of skin healing (inflammatory phase).

## Introduction

Skin injuries, or ulcers, are commonly observed in all age groups, but mostly among older adults, those who are immobile and those with severe acute illness or neurological deficits (1). Skin injuries can contribute to further deterioration of one’s general health (2). According to the Wound Healing Society, an estimated 2 to 3 million Americans are diagnosed with any type of chronic skin wound each year. Additionally, about 15% of older adults suffer from chronic wounds, including predominantly venous stasis ulcers, pressure ulcers (bedsores), and diabetic (neuropathic) foot ulcers (3).

One of the most relevant physiological factors related to skin injury pathogenesis is the presence of vascular dysfunction - such as chronic venous insufficiency and/or peripheral arterial occlusive diseases - and sustained hyperglycemia (4). However, hematologic autoimmune, malignant or primary skin diseases, genetic defects, the use of some medications and/or therapeutic procedures, and several exogenous factors can contribute to skin injury etiology (5,6). It is widely accepted that ischemia is a primary factor involved in skin injury genesis and evolution, but it is the blood reflow to ischemic areas that accelerates cell death due to an overproduction of reactive oxygen species (ROS). An increase in ROS levels that exceeds the antioxidant defenses of the skin leads to impairments in cellular biomolecular functions via a mechanism known as oxidative stress (7).

The skin injury healing process is complex and involves four main phases: homoeostasis, inflammation, proliferation (new tissue growth/matrix formation), and tissue remodeling (8,9). The inflammatory phase holds particular importance because an excessive inflammatory response could delay complete tissue healing. Therefore, research on the various techniques that improve the time to complete skin healing are of interest to the field of physiotherapy (10). Low-level laser therapy (LLLT) is a phototherapeutic modality that is widely used in clinical practice, and which employs different gas components. Distinct wavelengths are generated and result in different deep tissue targets (as major wavelengths reach deeper areas). The most common LLLTs used are helium/neon (HeNe, 630 ηm), aluminium/galium/indium/phosphide (Al-Ga-In-P, λ=660 ηm), gallium/aluminum/arsenide (GaAlAs, 820 and 830 ηm), and gallium/arsenide (GaAs, 904 ηm) (11,12).

Previous studies have shown that LLLT contributes to skin injury healing by accelerating wound contraction. This is achieved by increasing fibroblast and epithelium proliferation and also by improving collagen synthesis (13). The effects of LLLT on biological tissues are associated with light radiation absorption by photoacceptors or chromophores located in the mitochondria and hemoglobin of treated areas (11). Houreld et al. (14) reported an increase in cytochrome c oxidase activity as well as an increase in adenosine triphosphate (ATP) synthesis in fibroblasts irradiated with LLLT. Moreover, it was found that LLLT is effective at reducing necrotic areas in the skin possibly due to improvements in cytokine and growth/angiogenic factors’ recruitment, as well as enhancement of migration, proliferation, and differentiation of inflammatory cells (12).

Although the benefits of LLLT on skin injury healing have been reported previously, its biological mechanisms of action need to be fully understood. In this study, we first investigated the effects of LLLT on some hematological markers, such as number of blood constituents, to understand treatment effects on the development of the inflammatory response following a skin injury. Knowing that increased ROS formation is expected after skin injury, we also analyzed the effects of LLLT (Al-Ga-In-P, λ=660 ηm) on oxidative markers and antioxidant levels in injured/treated skin areas. Finally, to gain a better understanding of the results, the findings were divided into inflammatory (1 and 3 days) or regenerative (7, 14, and 21 days) phases.

## Material and Methods

### Animals

One hundred and twenty male adult Wistar rats weighing 250 to 300 g were obtained from the Animal Breeding Unit of the Federal University of Santa Maria (UFSM). Animals were allocated in cages with food and water *ad libitum* and maintained under standard conditions of temperature (22±1°C) and illumination (12-h light/dark cycle). The study protocol followed the ethical guidelines established by the Guide for Care and Use of Experimental Animals published by the National Institutes of Health (NIH publication No. 85-23, revised in 1996). All procedures outlined in this study were approved by the Ethics and Research Committee of UFSM (002/2014).

### Experimental design

Rats were divided into four experimental groups (n=120), Control (without injury or intervention), Sham (without skin injury with LLLT treatment), Skin injury (without LLLT treatment), and Skin injury + LLLT (with LLLT treatment). The animals were anesthetized with an intraperitoneal injection of ketamine (10%) and xylazine (2%) administered as 0.1 mL of solution per 100 g of body weight.

The dorsal region of each animal was shaved and disinfected with 70% alcohol. In the left region, between the infrascapular line and the tail, a circular area of skin of approximately 15 mm diameter and 5 mm deep was removed with a punch (Groups: Skin injury and Skin injury + LLLT) (15). The skin injuries were uniform in diameter, depth, and location. The right region, used as control (Groups: Control and Sham), was also anesthetized, shaved, and disinfected with 70% alcohol to ensure standardization (15). After the skin injury, animals were sub-divided in five subgroups according the analysis performed at 1 and 3 days (inflammatory phase) and 7, 14, and 21 days (regenerative phase) after injury.

### Laser application

Al-Ga-In-P laser (Ibramed, Brazil) with a wavelength of 660 ηm was applied in a single-point transcutaneous method with an energy density of 20 J/cm^2^, 30 mW power, and a time of 40 s. The first LLLT application was performed immediately after injury and repeated once a day every day until euthanasia.

### Tissue preparation and analysis

Rats submitted to the experimental procedures were euthanized with a lethal administration of thiopental (120 mg/kg, *ip*) at 1, 3, 7, 14, or 21 days after injury. Immediately after euthanasia, a sample of whole blood was collected by cardiac puncture in serum-separating tubes (5 mL) and another one in tubes containing EDTA (5 mL) (Vacutainer^®^, Becton Dickinson, USA).

A sample of skin of each animal (1 cm around the injury margin and deep into the subcutaneous fascia) was surgically excised and frozen in liquid nitrogen (-80°C) until the biochemical analysis.

Skin homogenates were prepared in cold saline (0.9% NaCl) in a 1:5 (weight:volume) proportion using an Ultra X80 stirrer (Ultra Stirrer, UK). Homogenates were centrifuged at 2,000 *g* in 4°C for 10 min to yield the low-speed supernatant (S1) fractions that were used for analysis.

#### Hematologic analysis

Blood samples collected into tubes containing EDTA (Vacutainer^®^) were used to determine total erythrocytes, hematocrit, hemoglobin, total leucocytes, and platelets using the automatic counter COULTER T890^®^ (Coulter Electronics, Inc., USA).

#### Measurement of lipid peroxidation (LPO)

LPO was quantified by malondialdehyde (MDA) formation in skin homogenates (16). In summary, skin homogenates were pre-incubated in a medium containing Tris-HCl as a buffer (10 mM, pH 7.4) for 60 min. Pre-incubation was ended by acetic acid buffer (pH 3.6) followed by addition of lauryl sodium sulphate (8.1% SDS) and thiobarbituric acid (0.6% TBA, pH 6.2). The mixture was incubated at 100°C for 60 min until color reaction.

#### Measurement of ROS production

ROS production in skin homogenates was determined by oxidation of reduced dichlorofluorescein (H_2_DCF-DA) (17). Briefly, skin homogenates were added to standard medium containing Tris-HCl as a buffer (10 mM, pH 7.4) and H_2_DCF-DA (1 mM) for 60 min in a condition without light. Fluorescence quantification was determined at 488 ηm for excitation and 525 ηm for emission, with slit widths of 3 ηm, in a spectrofluorometer (RF-5301 PC; Shimadzu, Japan) using oxidized dichlorofluorescein (DCF) as a standard.

#### Measurement of mitochondrial dehydrogenase activity (MTT reduction assay)

This assay is based on the ability of mitochondrial enzymes to metabolize MTT into formazan, a reaction that takes place only in functionally intact mitochondria, determined by methyl tetrazolium salt (MTT) reduction (18). Briefly, skin homogenates were pre-incubated with MTT at 30°C for 60 min. Dimethyl sulphoxide (1 mL DMSO) was added to extract colored components and measurements were made at 570 ηm. Results are reported in percent of the control (skin samples without injury and without LLLT) values.

#### Measurement of antioxidant enzyme activities

The antioxidant enzymes total superoxide dismutase (SOD) and catalase (CAT) were measured as described previously (19,20) in skin homogenates. For SOD analysis, the skin homogenate was added to a medium containing ethylenediamine tetraacetic acid (2 mM EDTA) and bicarbonate buffer (50 mM NaHCO_3_/Na_2_CO_3_, pH 10.3). Epinephrine (4 mM) was added to start the kinetic SOD activity for 5 min. The colored product of epinephrine degradation (that was inhibited by cellular SOD activity) was spectrophotometrically measured at 480 ηm. The SOD enzyme activity is reported in units of the enzyme activity per milligrams of protein. For CAT assay, skin homogenate was added to a medium containing potassium phosphate buffer (50 mM KH_2_PO_4_, 50 mM K_2_HPO_4_; pH 7.4). The kinetic analysis of CAT was started after hydrogen peroxide (1 mM H_2_O_2_) addition. The CAT activity was determined using the molar extinction coefficient 36 M^-1^cm^-1^ and the reaction was spectrophotometrically measured at 240 ηm.

#### Non-protein thiol (NPSH) measurement

NPSH levels were determined in skin homogenates according to the method proposed previously (21) with some modifications. Briefly, homogenate samples were precipitated with TCA (5%) (1:1) and subsequently centrifuged at 4,000 *g* for 10 min at 4°C. Supernatant fraction (500 μL) was added to a reaction medium containing K-phosphate buffer (0.25 mM, pH 7.4) and DTNB (1 mM). Spectrophotometrical measurements were made at 412 ηm. Results were calculated in relation to a standard curve constructed with GSH (reduced glutathione) at known concentrations and corrected by the protein content.

#### Protein measurement

Protein content was determined using bovine serum albumin (BSA) as the standard according to a previous study (17). Spectrophotometrical measurements were made at 595 ηm.

### Statistical analysis

Statistical analysis was performed using GraphPad (version 5.0 for Macintosh OSX, USA). The distribution of variables was tested using the Kolmogorov-Smirnov normality test. Significance was assessed by two-way analysis of variance (ANOVA), followed by Bonferroni test for *post hoc* comparison. Differences were significant when P<0.05.

## Results

### Effects of LLLT on hematological dynamics of the inflammatory phase

Hematocrit and hemoglobin demonstrated significant differences when exploring the injury × LLLT interaction (F_(1, 6)_=18.90, P<0.05). Three days following the skin injury, hematocrit (P<0.05, 95%CI=-15.70 to -3.963) and hemoglobin levels (P<0.05, 95%CI=-5.308 to -1.062) decreased compared to day 1 in the Skin injury group. Treatment with LLLT was effective against this decrease in hematocrit (P<0.05, 95%CI=4.496 to 16.24) and hemoglobin levels (P<0.05, 95%CI=1.129 to 5.374) (Table 1).

**Table 1:** Effects of low-level laser therapy (LLLT) on red and white blood cells in the inflammatory phase.

Hematological variable	Group (n, 4)	Collections		P value (ANOVA)			
		Day 1	Day 3	Treatment	Time	Interactions	
Hematocrit (g/dL)	Control	37.0±5.0	27.0±7.4^a^	0.1351	0.0045*	0.0048*	
	LLLT	37.6+1.2	37.5±3.1*				
Erythrocytes (x10^6^/µL)	Control	6.6±0.5	5.6±1.9	0.4264	0.5173	0.0187*	
	LLLT	6.2±1.1	7.04±0.7				
Hemoglobin (g/dL)	Control	12.4±1.6	9.2±2.3^a^	0.1213	0.0017*	0.0047*	
	LLLT	12.8±0.3	12.5±1.0*				
Leukocytes (x10^3^/µL)	Control	10.0±1.1	5.2±0.7^a^	0.0036*	0.0002*	0.1957	
	LLLT	14.7±0.5*	7.8±2.6^a^				
Platelets (x10^3^/µL)	Control	756.2±132.1	384.0±34.2^a^	0.2459	0.0003*	0.0054*	
	LLLT	566.2±64.0	466.2±13.4				

Data are reported as means±SE. *P<0.05 compared to Control group; ^a^P<0.05 compared to values of day 1 in the same experimental group (two-way ANOVA followed by *post hoc* Bonferroni test).

A significant interaction was found in erythrocytes in the inflammatory phase for injury × LLLT (F_(1, 6)_=10.22, P=0.0187); however, Table 1 shows that neither injury nor treatment with LLLT caused a significant difference in erythrocyte count.

In the early stages of wound healing, the leucocytes presented a significant main effect of time (F_(1, 6)_=21.34, P=0.0036) and LLLT (F_(1, 6)_=66.53, P<0.001). LLLT was associated with increased leucocyte count on the first day compared to the Skin injury group (P<0.05, 95%CI=1.961 to 7.439). Nevertheless, on the third day, the Control group and the LLLT group demonstrated a reduction in leucocyte count compared to the first day (P<0.05, 95%CI=1.805 to 7.862 and 95%CI=3.905 to 9.962, respectively).

An examination of the platelet count revealed a significant injury × LLLT interaction (F_(1, 6)_=18.06, P=0.0054). The Skin injury group had a significant decrease in platelet levels on the 3rd day compared to the first day (P<0.05, 95%CI=238.8 to 509.7), and LLLT was effective against this decrease, as it helped to maintain these levels (Table 1).

### Effects of LLLT on skin oxidative damage in the inflammatory phase

**Figure 1:**
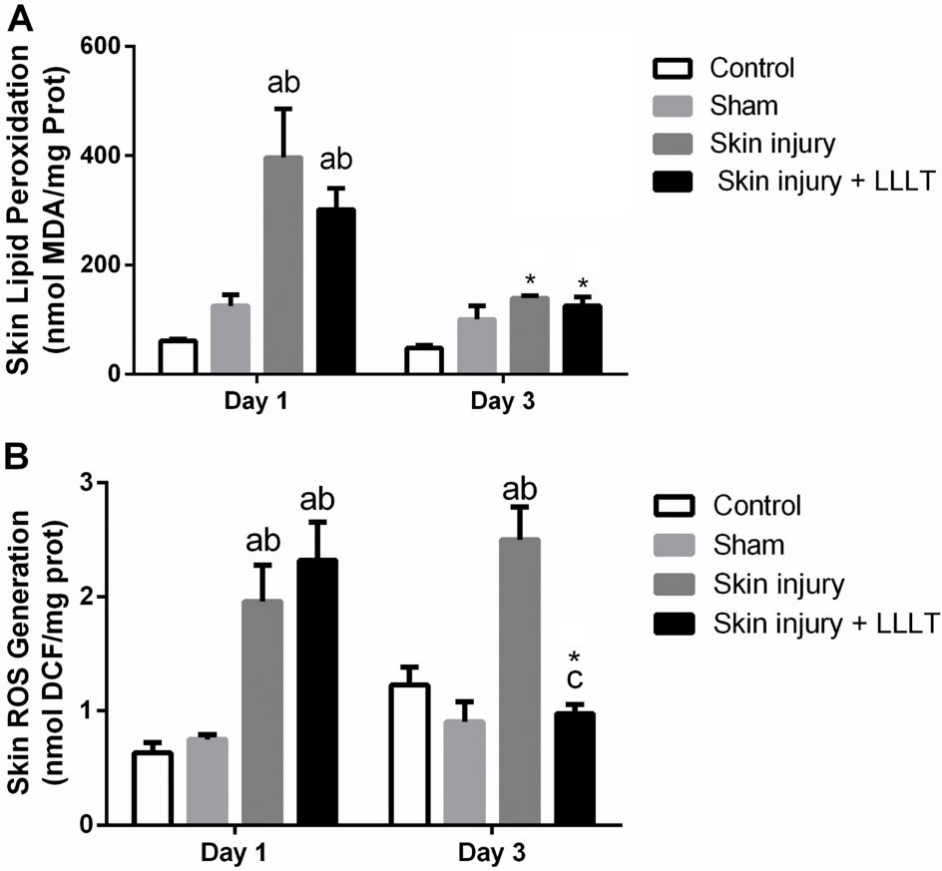
Effects of low-level laser therapy (LLLT) (660 ηm) on skin oxidative damage markers in the inflammatory phase. **A**, Lipid peroxidation (TBARS) and (**B**) reactive species production (oxidized DCF) levels. Data are reported as means±SE. *P<0.05 compared to day 1 in the same experimental group; ^a^P<0.05 compared to Control group (P<0.05); ^b^P<0.05 compared to Sham group; ^c^P<0.05 compared to Skin injury group (ANOVA followed by *post hoc* Bonferroni test). ROS: reactive oxygen species.

Figure 1A shows that thiobarbituric acid reactive substances (TBARS) had a significant injury × laser interaction (F_(3, 20)_=8,427, P<.0008). The Skin injury group had higher lipid peroxidation levels on the first day compared to the Control (P<0.0001, 95%CI=239.3 to 498.4) and Sham groups (P<0.0001, 95%CI=175.5 to 434.6). Treatment with LLLT decreased lipid peroxidation levels. In addition, on the third day, Skin injury and Skin injury + LLLT showed a reduction in the lipid peroxidation levels compared to the first day (P<0.001, 95%CI=-399.0 to -115.4; P<0.05, 95%CI=-317.8 to -34.28, respectively).

ROS levels showed a significant injury × laser interaction (F_(3, 19)_=7.731, P=0.0014). On day 1, the Skin injury group had higher mitochondrial ROS levels compared to those of the Control group (P<0.001, 95%CI=0.5099 to 2.142) and the Sham group (P<0.01, 95%CI=0.3919 to 2.024); LLLT was not effective against this increase. Nevertheless, LLLT significantly reduced the ROS levels on day 3 compared to day 1 (P<0.05, 95%CI=-2.71 to -0.4206), and it was found to be effective in relation to the increase resulting from the skin injury (P<0.001, 95%CI=-2.382 to -0.6698) (Figure 1B).

### Effects of LLLT on mitochondrial dehydrogenase activity in the inflammatory phase

**Figure 2:**
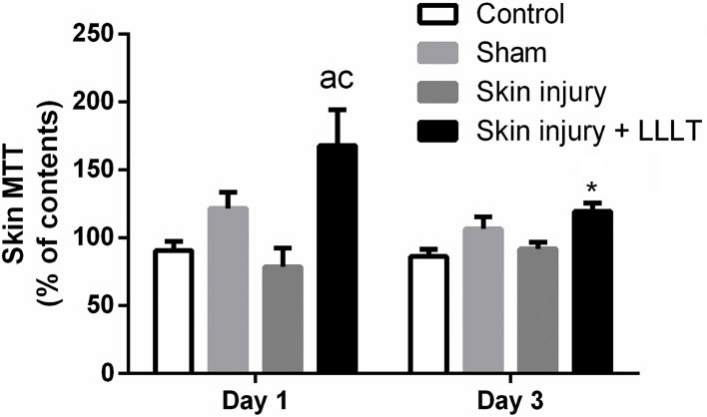
Effects of low-level laser therapy (LLLT) (660 ηm) on skin cell viability (MTT reduction levels) in the inflammatory phase. Data are reported as means±SE. *P<0.05 compared to day 1 in the same experimental group; ^a^P<0.05 compared to Control group (P<0.05); ^c^P<0.05 compared to Skin injury group (ANOVA followed by *post hoc* Bonferroni test).

Dehydrogenase activity showed a significant main effect of treatment (F_(3, 24)_=7.961, P=0.0007). On the first day, the Skin injury + LLLT group had increased dehydrogenase activity compared to the Control (P<0.001, 95%CI=28.47 to 126.1) and Skin injury groups (P<0.0001, 95%CI=40.62 to 138.3). However, by day 3, LLLT was effective at maintaining this increase compared to the activity noted on day 1 (P<0.05, 95%CI=-92.23 to -4.938) (Figure 2).

### Effects of LLLT on skin enzymatic and non-enzymatic antioxidants in the inflammatory phase

**Figure 3:**
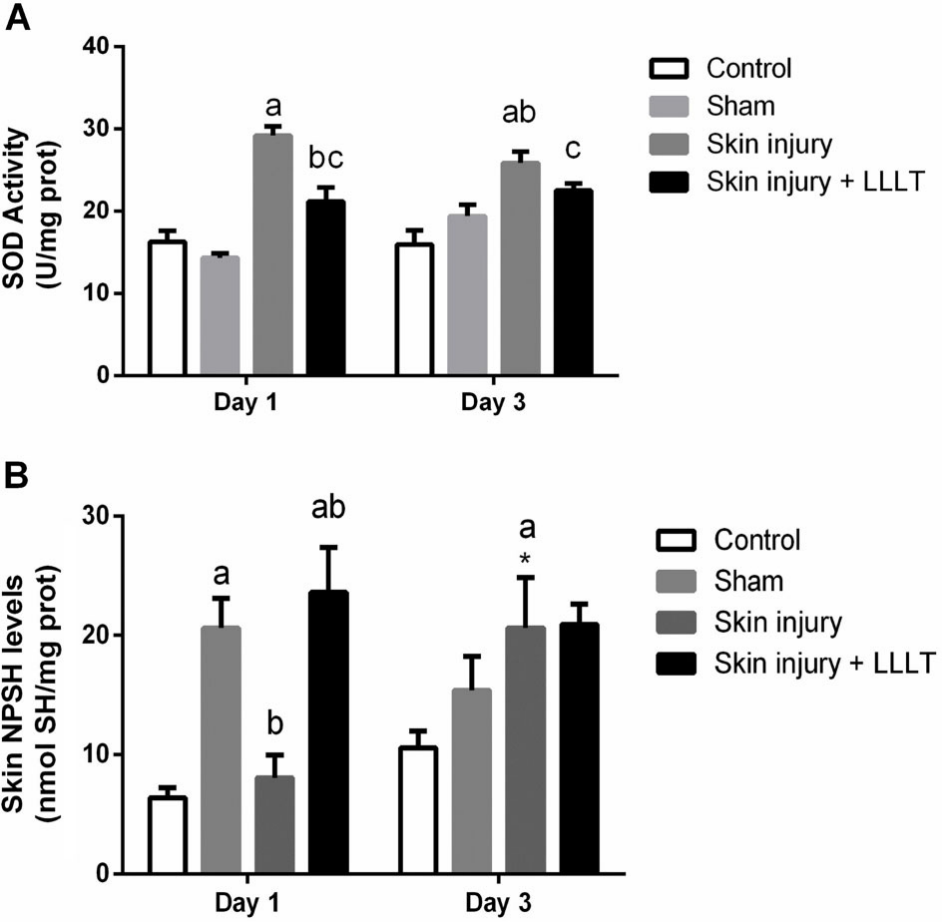
Effects of low-level laser therapy (LLLT) (660 ηm) on skin antioxidants in the inflammatory phase. **A**, Superoxide dismutase enzyme activity (SOD) and (**B**) non-protein thiol levels (NPSH). Data are reported as means±SE. *P<0.05 compared to day 1 in the same experimental group; ^a^P<0.05 compared to Control group (P<0.05); ^b^P<0.05 compared to Sham group; ^c^P<0.05 compared to Skin injury group (ANOVA followed by *post hoc* Bonferroni test).

SOD activity results revealed a significant injury × laser interaction (F_(3, 20)_=3.452, P=0.036). On the first day, the Skin injury group had increased SOD activity compared to the Control (P<0.05, 95%CI=7.804 to 18.10), Sham (P<0.05, 95%CI=9.727 to 20.03), and Skin injury + LLLT groups (P<0.05, 95%CI=-13.20 to -2.896). The Skin injury group maintained greater SOD activity during 3 days compared to the Control (P<0.05, 95%CI=4.799 to 15.10) and Sham groups on the first day (P<0.05, 95%CI=1.321 to 11.62). On the third day, LLLT promoted an increase in SOD activity compared to the Control group (P<0.05, 95%CI=1.414 to 11.71) (Figure 3A).

Figure 3B shows the effect of the LLLT-altered non-protein thiol levels in the skin, which demonstrated a significant main effect of laser (F_(3, 20)_=4.288, P=0.0172) and treatment (F_(3, 20)_=10.67, P=0.0002). There was no change in the NPSH levels in the Skin injury group on the first day compared to the Control group, although LLLT promoted NPSH level increases in the Sham (P<0.001, 95%CI=-22.87 to -2.238) and Skin injury + LLLT groups (P<0.001, 95%CI=5.240 to 25.88) compared to the Skin injury group. After 3 days, the Skin injury group showed an increase in NPSH levels compared to day 1 (P<0.05, 95%CI=2.019 to 23.16).

### Effects of LLLT on the hematological dynamics of the regeneration phase

In the regeneration phase, erythrocyte counts presented a significant main effect of time (F_(1, 6)_=13.48; P=0.0009). Similarly, hematocrit and hemoglobin demonstrated significant main effects of time (F_(1, 6)_=20.58, P<0.001; F_(1, 6)_=26.41, P<0.001, respectively) (Table 2). Neither the Skin injury group nor Skin injury + LLLT group presented significant differences.

**Table 2:** Effects of low-level laser therapy (LLLT) on red and white blood cells in the regenerative phase.

Hematological variable	Group (n, 4)	Collections			P value (ANOVA)		
		Day 7	Day 14	Day 21	Treatment	Time	Interactions
Hematocrit (g/dL)	Control	31.4±4.8	32.5±3.7	39.4±0.4	0.4767	0.0001*	0.3567
	LLLT	34.7±0.9	31.6±3.3	40.3±1.1			
Erythrocytes (x10^6^/µL)	Control	6.0±0.9	6.1±0.7	7.2±0.02	0.6893	0.0009*	0.6694
	LLLT	6.3±0.3	5.9±0.6	7.4±0.1			
Hgb (g/dL)	Control	10.2±1.7	11.0±1.2	13.3±0.2	0.242	<0.0001*	0.136
	LLLT	11.8±0.4	11.1±0.4	13.4±0.3			
Leukocytes (x10^3^/µL)	Control	6.4±0.3	8.1±1.9	9.5±1.1	0.8776	0.0181*	0.3888
	LLLT	7.4±0.9*	7.7±2.9	8.9±0.7			
Platelets (x10^3^/µL)	Control	538.5±38.8	746.5±88.5^a^	679.5±3.6^a^	0.0337*	<0.0001*	0.0389*
	LLLT	488.2±19.6	597.5±82.8*^a^	654.2±16.7^a^			

Data are reported as means±SE. *P<0.05 compared to Control group; ^a^P<0.05 compared to values of day 7 in the same experimental group (two-way ANOVA followed by *post hoc* Bonferroni test).

Leucocyte counts revealed a significant main effect of time (F_(1, 6)_=5.902, P=0.0162) in the regeneration phase. The Skin injury group presented a reduction in leucocyte levels within 7 days, and LLLT was effective against this decrease (P<0.05, 95%CI=1.805 to 7.862) (Table 2).

Platelet counts showed a significant injury × laser interaction (F_(1, 6)_=3.306, P=0.0038). The Skin injury group had increased platelet levels on days 14 (P<0.05, 95%CI=53.35 to 228.6) and 21 (P<0.05, 95%CI=120.4 to 295.6) compared to day 7. The LLLT increased the platelet levels at 14 days compared to 7 days (P<0.05, 95%CI=21.60 to 196.9), as well as at 21 days compared to 14 days (P<0.05, 95%CI=78.35 to 253.6). Thus, LLLT promoted a gradient increase in platelet levels compared to the Skin injury group at 14 days (P<0.05, 95%CI=-248.1 to -49.94) (Table 2).

### Effects of LLLT on skin oxidative damage in the regeneration phase

**Figure 4:**
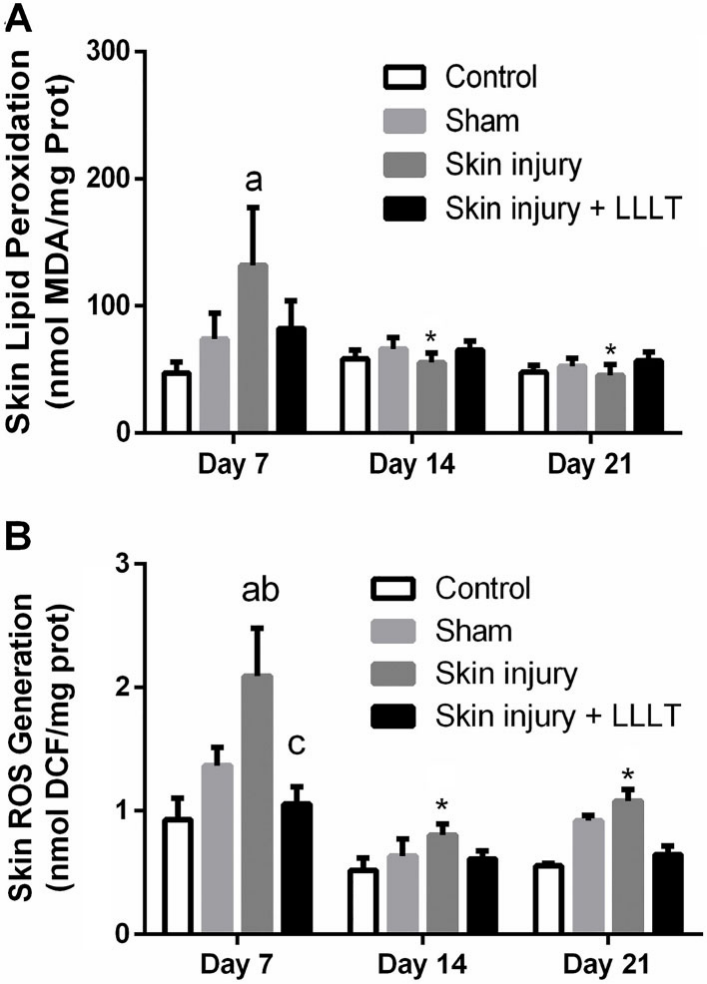
Effects of low-level laser therapy (LLLT) (660 ηm) on skin oxidative damage markers in the regenerative phase. **A**, Lipid peroxidation (TBARS) and (**B**) reactive oxygen species (ROS) production (oxidized DCF) levels. Data are reported as means±SE. *P<0.05 compared to day 7 in the same experimental group; ^a^P<0.05 compared to Control group (P<0.05); ^b^P<0.05 compared to Sham group; ^c^P<0.05 compared to Skin injury group (ANOVA followed by *post hoc* Bonferroni test).

In the regenerative phase, TBARS showed a significant effect of time (F_(3, 20)_=3.924, P*=*0.0277). On the seventh day, the Skin injury group demonstrated a sustained increase in lipid peroxidation levels compared to the Control group (P<0.05, 95%CI=19.09 to 150.2), and LLLT was effective against this increase (P<0.001, 95%CI=-1.627 to -0.4491) (Figure 4A).

Only at day 14 did the Skin injury group demonstrate a reduction in lipid peroxidation levels compared to day 7 (P<0.001, 95%CI=-136.7 to -15.99). This was maintained until day 21 (P<0.001, 95%CI=-136.7 to -15.99) (Figure 4A).

The ROS levels showed a significant main effect of time (F_(3, 19)_=25.26, P=0.0001) and LLLT (F_(3, 19)_=10.21; P=0.0003). The Skin injury group showed an increase in ROS levels compared to the Control (P<0.0001, 95%CI=0.5767 to 1.755) and Sham groups (P<0.001, CI=0.1374 to 1.315), and LLLT was effective against this increase (P<0.001, 95%CI=-1.627 to -0.4491). Furthermore, the Skin injury group showed a reduction in ROS levels on day 14 compared to day 7 (P<0.001, 95%CI=-1.627 to -0.4491); this effect was sustained until 21 days (P<0.001, 95%CI=-1.544 to -0.4829) (Figure 4B).

### Effects of LLLT on mitochondrial dehydrogenase activity in the regeneration phase

**Figure 5:**
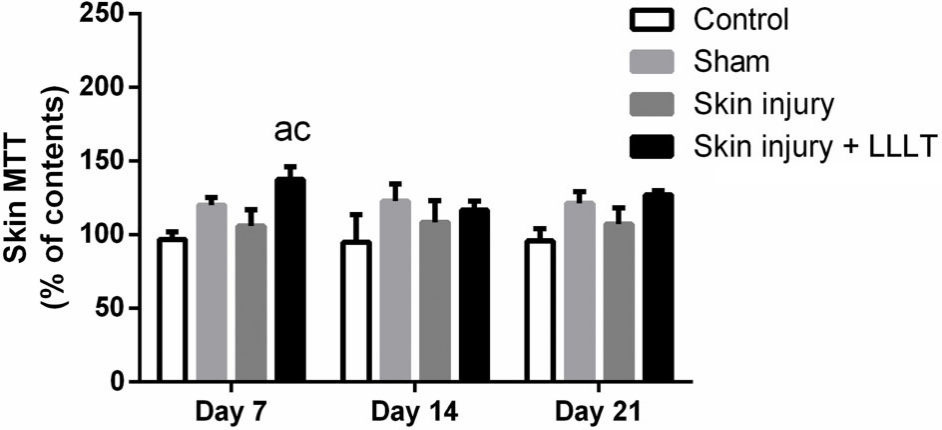
Effects of low-level laser therapy (LLLT) (660 ηm) on skin cell viability (MTT reduction levels) in the regenerative phase. Data are reported as means±SE. ^a^P<0.05 compared to Control group (P<0.05); ^c^P<0.05 compared to Skin injury group (ANOVA followed by *post hoc* Bonferroni test).

Dehydrogenase activity showed a significant main effect of LLLT (F_(3, 24)_=3.062, P=0.0474). Skin injury + LLLT had increased MTT levels compared to the Control (P<0.05, 95%CI=1.500 to 80.07) and Skin injury groups (P<0.0376, 95%CI=1.500 to 80.07) (Figure 5).

### Effects of LLLT on skin enzymatic and non-enzymatic antioxidants in the regeneration phase

SOD activity showed a significant main effect of LLLT (F_(3, 20)_=4.338, P=0.0165). On day 7, the Skin injury + LLLT group demonstrated decreased SOD activity compared to the Sham group (P<0.05, 95%CI=-8.261 to -0.8038).

**Figure 6:**
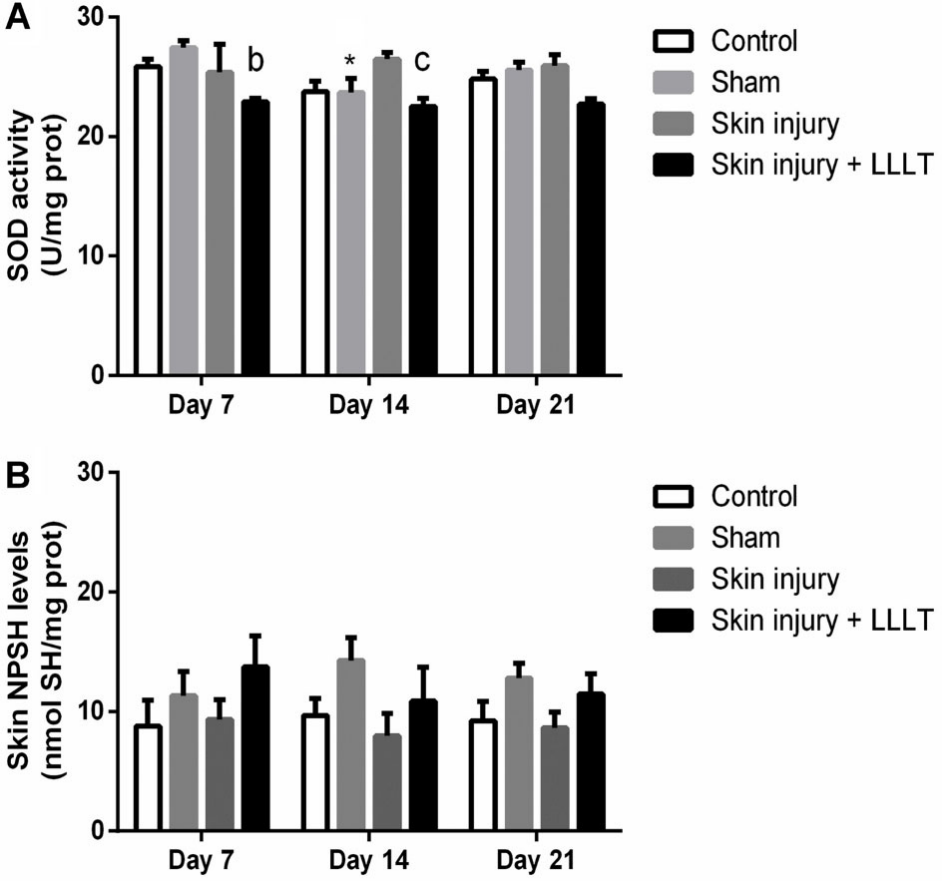
Effects of low-level laser therapy (LLLT) (660 ηm) on skin antioxidants in the regenerative phase. **A**, Superoxide dismutase enzyme activity (SOD) and (**B**) non-protein thiol levels (NPSH). SOD values are reported in units of enzyme activity per mg of protein. Data are reported as means±SE. *P<0.05 compared to day 7 in the same experimental group; ^b^P<0.05 compared to Sham group; ^c^P<0.05 compared to Skin injury group (ANOVA followed by *post hoc* Bonferroni test).

LLLT alone (Sham group) exhibited decreased SOD activity at 14 days compared to day 7 (P*<*0.05, 95%CI=-6.677 to -0.8078). The Skin injury + LLLT group also showed a decrease in this enzymatic activity compared to the Skin injury group on day 14 (P<0.05, 95%CI=-7.663 to -0.2053) (Figure 6A).

Neither skin injury nor treatment promoted a significant change in non-protein thiol levels in the skin (Figure 6B).

## Discussion

We explored the effect of LLLT on its ability to reduce the oxidative stress in skin homogenate and to alter the blood parameters in rats. We used LLLT, a well-known intervention for skin injury, to better understand the effects in different phases of cellular healing. To the best of our knowledge, this is the first study to demonstrate that LLLT maintained hematocrit and hemoglobin levels until the third day of treatment (leukocyte levels increased on the first day). In fact, LLLT increased the total leukocyte levels compared to the control until day 3. This result was in accordance with the observations made by Boschi et al. (22), who conducted a study using LLLT (21 J/cm^2^) for 1, 2, and 3 h following carrageenan injection. Machneva et al. (23) also reported an increase in leukocyte activity in association with red laser radiation in animals injected with lipopolysaccharides. Leukocytes, edema, and other hematological parameters are considered the key players of the inflammatory response (24). Taken together, the hematological results suggested that LLLT anticipated inflammatory cell recruitment to the injured area, thus enhancing the healing process in Wistar rats.

Energy absorption is the primary mechanism that enables light from the laser to produce biological effects in tissue. Light absorption is dependent on wavelength, and the main tissue chromophores - such as hemoglobin and melanin - strongly absorb wavelengths shorter than 600 nm (11). LLLT demonstrated the efficiency of therapeutic light, as it was able to maintain hematocrit and hemoglobin levels in the inflammatory phase.

The specific mechanism underlying the action of light at the cellular level is based on photobiological reactions, which mediate the biological effects. A photobiological reaction is when photoreceptor molecules absorb a specific wavelength of light (11). A previous study reported that LLLT increases ATP synthesis after 3 min of irradiation (25). Karu et al. (26) reported increases in ATP synthesis induced by LLLT in human cervical cancer (HeLa) cells, but only after 20-25 min of irradiation. Other studies have reported the dose-dependent effects of LLLT on mitochondria, such as increases in mitochondrial membrane potential (MMP) (27) and in mitochondrial complex IV activity (cytochrome c oxidase) (14). In fact, the primary reactions underlying light activity on the photoreceptors have not yet been clearly established, but some hypotheses have been put forward (11). It is believed that mitochondrial complex IV activation is the basic biological mechanism responsible for the effects of LLLT, as it is considered the major site of light issued by LLLT absorption (28–30).

Our findings demonstrated that the effects of LLLT on mitochondrial activity showed significant increases in dehydrogenase enzyme activity, mainly located in mitochondria (18) (MTT levels) in inflammatory and regenerative phases (from days 1 to 7). This result is aligned with the findings of Volpato et al. (31), who reported an increase in MTT levels with LLLT treatment in fibroblasts after 72 h. Considering that an increased reduction in MTT levels is associated with higher dehydrogenase enzyme activity in the mitochondria, it is possible that LLLT may improve the mitochondrial activity of skin-treated areas.

Mitochondria are primarily involved in the regulation of cell proliferation, ATP generation, cell death, and metabolism (26). Nevertheless, this organelle was recently identified as a central factor involved in the control of innate immunity and the inflammatory response, while the intrinsic dynamicity of the mitochondrial compartment plays a central role in proinflammatory signaling (32). In addition, previous studies already associated LLLT with increased electron flux through the mitochondrial transport chain (33,34). It is interesting to note that the increase in dehydrogenases enzyme activities was observed only when LLLT was associated with skin injury.

The importance of ATP in the inflammation phase has been established. Specifically, ATP released from stressed cells functions as a “danger” and “find-me” signal for phagocytes to migrate to the damaged tissue (35). During the inflammation phase, ATP may be released by activated platelets and leukocytes. In the same way, the mitochondria constitute a major source of ROS production, while mitochondrial complex activity helps in the electron escape (36). Following the application of LLLT, cytochrome c oxidase becomes electrically charged, from which it alters its redox status and causes an increase in electron transfer in the respiratory chain. This then enables an increase in the production of superoxide anion (11).

In fact, skin injury significantly increased oxidative damage markers (lipid peroxidation) until day 7, and it also increased ROS generation in the inflammatory and regenerative phases. ROS overproduction triggers oxidative damage (including lipid peroxidation) and alters the mitochondrial membrane potential that results in extensive oxidative damage and cell death. In addition, the tissue repair process consists of several interdependent phases: degeneration, inflammation, regeneration, fibrosis/scar formation, and remodeling (8,9). Evidence has suggested that uncontrolled ROS generation plays an important role in the formation of fibroses. This phase usually occurs around 14 days after injury, reaching its peak at 21 days, as characterized by the synthesis and deposition of collagen (37).

However, LLLT exhibits this profile only during the inflammatory phase, and mostly on the first day. LLLT was reported to produce a change in the cell redox potential while increasing ROS generation in the inflammatory phases (37). This shift in redox state induced the activation of numerous intracellular signaling pathways, such as nucleic acid synthesis, protein synthesis, enzyme activation, and cell cycle progression (34). Furthermore, an increase in ROS levels and, consequently, in oxidative pathways seems to be crucial for the effects of LLLT on mitochondrial activity in the inflammatory phases (32). Our results suggested that LLLT can use the increase in ROS as a cellular signaling pathway to thereby start the tissue-repair process.

Overall, the SOD levels significantly changed in the skin lesions. There was a significant decrease in the non-protein-SH group levels, caused by the injury during the acute inflammatory phase. However, by day 3, the antioxidant system showed a skin injury-promoted increase. Treating the lesion with LLLT decreased SOD activity compared to the skin injury in the inflammatory phase. One possible reason for this observed antioxidant depletion may be that the antioxidant system increased the circulating ROS levels. LLLT effectively reduced the decrease in non-protein-SH group levels, and the effects of LLLT on non-protein-SH group levels cemented our understanding of the treatment’s actions on cellular systems. Moreover, previous studies have further established that LLLT decreases SOD activity (38).

### Conclusion

Taken together, these results led us to suggest that LLLT accelerated skin tissue repair via a mechanism that modulated increased ROS levels and lipid peroxidation, especially in the early stages of skin injury healing (i.e., during the inflammatory phase). Considering that ROS play a signaling role in cell survival and proliferation, we hypothesize that LLLT can be used as a cellular signaling pathway to increase ROS. In this way, LLLT can decrease the amount of time needed for tissue repair. In order to confirm this hypothesis, further studies are needed to explore the effects of LLLT on mitochondrial activity and the signal transcription of skin cells. This will help elucidate the therapeutic mechanisms of action, while also amplifying and improving clinical applications of this therapeutic approach.
